# SPATS2 is correlated with cell cycle progression and immune cells infiltration in hepatocellular carcinoma

**DOI:** 10.1186/s12876-022-02633-y

**Published:** 2023-01-11

**Authors:** Jing Lin, Jia Yan, Xiu ling Deng, Chang shan Wang, Hai sheng Wang

**Affiliations:** 1 College of Life Science, Inner Mongolia University, Hohhot, Inner Mongolia China; 2 School of Basic Medical, Inner Mongolia Medical University, Hohhot, Inner Mongolia China

**Keywords:** Hepatocellular carcinoma, SPATS2, Cell cycle, Immune infiltration

## Abstract

The spermatogenesis associated serine rich 2 (SPATS2) is a member of RNA-binding protein in which the abnormal expression is linked with carcinogenesis in serval types of cancer. However, there is no systematic study on the differential expression, prognostic significance, epigenetic regulation, immune infiltration of SPATS2 in hepatocellular carcinoma (HCC). In the present study, we investigated the expression, prognosis, epigenetic regulation, and immune cell infiltration of SPATS2 in HCC. We found that the elevated expression of SPATS2 was unfavorably associated with the clinical pathological stage and prognosis. Functional enrichment analysis revealed that SPATS2 is associated with cell cycle, apoptosis and cancer cell metastasis processes in HCC. Our results confirmed that knockdown of SPATS2 will affect cell cycle, apoptosis and invasion of HCC cell lines. Moreover, the expression of SPATS2 is upregulated by epigenetic regulation, including DNA methylation, m6A and histone modification in HCC. In addition, SPATS2 expression was positively correlated with immune cell infiltration or expression of immune related gene markers in HCC. Taken together, our data demonstrated that SPATS2 is associated with progression and immune infiltration, and could serve as a prognostic biomarker for HCC. In conclusion, these results highlight the potential of SPATS2 to be used as a therapeutic target for HCC.

**Supplementary Information:**

The online version contains supplementary material available at 10.1186/s12876-022-02633-y.

## Introduction

Hepatocellular carcinoma (HCC) is the most common primary liver malignancy [1, 2]. It is the sixth most commonly diagnosed malignancy and the third leading cause of cancer-related mortality worldwide [2, 3]. In the past few decades, new therapeutic strategy, such as molecular targeted therapies and immunotherapy, have been developed and clinically evaluated with interesting results in HCC [4]. Remarkably, the combination of immune checkpoint inhibitors and VEGF inhibitors (Atezolizumab plus Bevacizumab) have been tested and approved for the treatment of advanced HCC [5]. Although many new therapeutic strategies have been widely used and improved, the long-term survival rate of HCC is still unsatisfactory [5]. Therefore, it is critical to reveal the carcinogenesis mechanisms and explore new potential therapeutic targets to improve survival of HCC patients.

The spermatogenesis associated serine rich 2 (SPATS2) is a cytoplasmic RNA-binding protein, which is mainly expressed in adult testis and slightly expressed in liver and other tissues [6, 7]. It has been reported that SPATS2 serves a tumorigenic role in several cancers, such as esophageal squamous cell carcinoma, colorectal cancer, prostate cancer, and HCC [6–9]. SPATS2 is identified as a novel diagnostic biomarker in squamous cell carcinoma [10]. Moreover, SPATS2 promotes lncRNA SNHG5-mediated survival of colorectal cancer cells through pro-proliferative and anti-apoptotic effect [8]. Recently, SPATS2 is reported to be a diagnostic and prognostic biomarker in liver cancer [11]. SPATS2 negatively regulates by miR-145-5p and results in promoting hepatocellular carcinoma progression through regulating cell cycle [6]. Moreover, it is still involved in the proliferation and invasion of HCC cells through TRIM44-STAT3 signaling pathway [12]. Therefore, SPATS2 may be a potential liver cancer marker. However, the function of SPATS2 in HCC needs to be further clarified.

Extensive studies have illustrated that the interplay between cancer cells and the tumor microenvironment (TME) plays a significant role in ineffective treatment and a poor prognosis of cancer [13]. The main cellular components in the HCC TME include immune cells, fibroblasts, macrophages, and cancer stem cells [14]. The levels of these cells and related molecules are crucial for the tumor cell survival, growth, proliferation, epithelial–mesenchymal transition, metastasis and tumor immune escape [15]. Therefore, finding and understanding the function of TME-related molecules are essential for the effective management and precision anticancer therapies [15]. In recent years, immunotherapy has brought beneficial effects in a variety of solid tumors. In hepatocellular carcinoma (HCC) patients, it was only a subgroup of HCC patients responded to immunotherapy [16, 17]. It is important to explore new prognostic biomarkers and potential predictors of immunotherapeutic response for HCC.

In this study, we assessed the diagnostic and prognostic values of SPATS2 in HCC. Moreover, the increased mRNA level and reduced methylation level of SPATS2 were associated with poor survival of patients with HCC. It is a prognostic biomarker and involved in cell cycle, apoptosis, and metastasis of HCC progressions. In addition, SPATS2 expression and its methylation were associated with the immune infiltration levels of different immune cell subtypes in HCC. Therefore, SPATS2 is likely a potential prognostic and diagnostic biomarker related to immune infiltration in HCC microenvironment. This will be benefit to improve the prognostic prediction and personalized treatment management of immunotherapy in HCC.

## Materials and methods

### Gene expression analysis

For differential expression analysis, GEPIA2 was used to detect the expression of SPATS2 based on the TCGA (The Cancer Genome Atlas) and GTEx (Gene Tissue Expression) databases. The expression of SPATS2 in different tumor stages and grades was confirmed using UALCAN (The University of Alabama at Birmingham Cancer data analysis Portal) database based on the TCGA_LIHC (Liver Hepatocellular Carcinoma) data. In addition, the protein expression level of SPATS2 was further verified by immunohistochemical staining in tumor tissues from patients with HCC using the HPA (Human Protein Atlas) database.

### Kaplan–Meier survival analysis

The survival data of patients with liver cancer was derived from the TCGA database. Kaplan–Meier survival analysis was performed to determine the predictive value of SPATS2 in LIHC, including overall survival (OS), disease-specific survival (DSS), disease-free survival (DFS), and progression-free survival (PFS). Moreover, Kaplan–Meier survival analysis was completed to confirm the OS of patients with HCC based on the SPATS2 expression and the infiltration levels of different immune cell subtypes. The results of *p* < 0.05 were considered to be statistically significant.

### Analysis hub genes of SPATS2 co-expressed in HCC

We obtained the differentially expressed genes related to SPATS2 in LIHC using the LinkedOmics database. These SPATS2 related genes were annotated using Gene Ontology (GO) analysis. Moreover, the Kyoto Encyclopedia of Genes and Genomes (KEGG) pathway enrichment analysis was performed via the gene set enrichment analysis (GSEA) model in LIHC. Additionally, The GSCA (Gene Set Cancer Analysis) database was used to analyze the pathway enrichment of the co-expressed gene set of SPATS2. The Cytoscape software was used to determine its hub genes in HCC base on the proteins interaction networks from String database.

### Cell culture and transfection

The normal liver cell line (LO_2_) and HCC cell lines (HepG2 and MHCC97-H) were obtained from the Type Culture Collection of the Chinese Academy of Sciences (Shanghai, China). These cells were cultured in Dulbecco’s modified Eagle’s medium (DMEM) (Gibco, USA) containing 10% FBS (Gibco, USA), 10 U/ml penicillin, and 10 mg/ml streptomycin (Sigma, USA). The cells were grown in a sterile incubator with a humidified atmosphere containing 5% CO_2_ at 37 °C.

The specific siRNA targeted to *SPATS2* was synthesized by GenePharma (Shanghai, China). Empty vector was utilized as negative control. Lipofectamine 2000 (ThermoFisher Scientific, Waltham, USA) was used for the transfection of all these vectors and reagents into cells. All transfected cells were collected for subsequent use after 48 h later.

### Cell apoptosis assay

We collected 1 × 10^6^ transfected HCC cells and treated with buffering from FITC-Annexin V apoptosis kit (Sungenebiotech, China), which includes 5 μl Annexin V-FITC and 5 μl PI for 20 min at room temperature in a dark environment. Then, the rate of cell apoptosis was obtained from flow cytometry (Beckman Coulter, Inc., Brea, USA).

### Cell cycle assay

The transfected HCC cells were fixed with 70% pre-chilled ethanol overnight. They were washed with PBS and stained with 20 μL Propidium iodide (Sigma, USA). Then, the rate of different cell cycle was analyzed by flow cytometry (Beckman Coulter, Inc., Brea, USA).

### Cell invasion assay

The transfected HCC cells (1 × 10^5^) were suspended in 200 µl of serum-free medium and seeded into the upper chamber, while 600 µl of medium containing 10% FBS was added to the lower chamber. After incubation for 48 h at 37 °C, the remaining cells on the upper surface were removed with cotton swabs. The membranes were fixed in methanol and stained with 0.5% crystal violet. Cells on the lower surface of the membrane were counted in randomly selected fields.

### Epigenetic analysis

DNA methylation of SPATS2 at TSS1500 sites and the prognostic value of this site in HCC were confirmed by MethSurv database, a web tool to perform multivariable survival analysis using DNA methylation data. The methylation level of the SPATS2 promoter region was determined via UALCAN database based on the TCGA_LIHC data.

### Immune infiltration analysis

The TIMER (Tumor Immune Estimation Resource) database and the GSCA was used to calculate the abundance of tumor infiltrating immune cells (TIICs) in tumor tissues of LIHC [18, 19]. TIMER was used to analyze the mRNA expression-related infiltration of different types of immune cells in liver cancer. The relationships between SPATS2 mRNA levels and the infiltration of different immune cell subtypes were performed using the immune module of GSCA database. The correlations of SPATS2 expression with the critical immunomodulators in liver cancer were evaluated using TISIDB, which is an integrated repository portal for tumor-immune system interactions [20]. The *p* < 0.05 results were considered to be statistically significant.

### Statistical analysis

All the experiment was independently repeated three times. Data were summarized as the mean ± SD (Standard Deviation). Partial results were analyzed using GraphPad Prism 8.0 software. The Student’s *t*-test was used to analyzed the different between two groups, while one-way ANOVA wase performed to evaluate the statistical significance among multiple groups. The results were considered to be statistically significant when the value of *p* was < 0.05.

## Results

### Relationships between the clinicopathological and prognostic features of SPATS2 in HCC

**Fig. 1:**
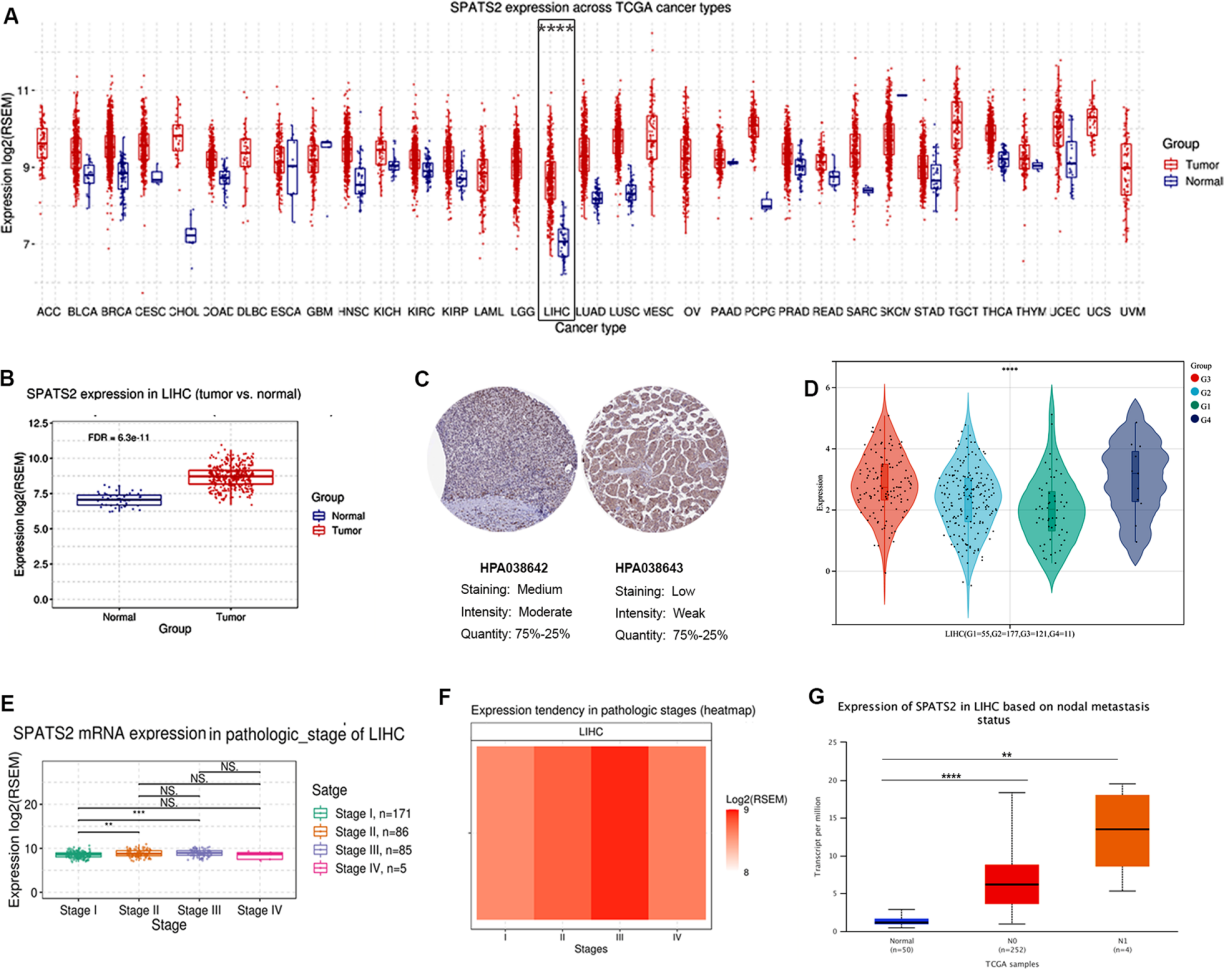
Clinical implication of SPATS2 in LIHC. **A** The expression of SPATS2 in pan cancer based on the TCGA results. SPATS2 is significantly upregulated in LIHC. *p* value is1.9e−39. **B** The expression of SPATS2 in LIHC and paired normal tissues by box plot via GSCA database. *p* value is 1.2e−12. **C** Immunohistochemical analysis of SPATS2 protein expression in HCC tissues based on the protein expression results of HPA database. **D**–**G** Correlation of SPATS2 with clinical information of HCC. **D** SPATS2 is correlated with tumor grades of LIHC. **E**, **F** SPATS2 is associated with pathologic stage of LIHC. It is upregulated in stage III of patients with HCC. *p* value is 3.89e−3. **G** Expression of SPATS2 is higher in metastasis status of LIHC. *p* value is 4.018200e−02 and 1e−12, especially. *p < 0.05, **p < 0.01, ***p < 0.001

To further evaluate the functions of SPATS2, we assessed the profiles of SPATS2 expression across various types of cancers based on the results in TCGA databases. We found that SPATS2 was remarkably upregulated in LIHC (Fig. 1A). GSCA result also showed that SPATS2 expression was significantly increased in tumor tissues of LIHC than that in normal tissues (Fig. 1B). Moreover, SPATS2 protein level was upregulated in LIHC based on the protein expression results of HPA database in liver cancer (Fig. 1C). Following, the relationships of SPATS2 expression with the clinicopathological parameters of patients with HCC were assessed by UALCAN database. As shown in Fig. 1D, a significant correlation was found between SPATS2 expression and tumor grades of patients with HCC. Moreover, it is associated with pathologic stage of LIHC, especially in the stage III (Fig. 1E, F). Additionally, SPATS2 was elevated in the nodal of metastasis status in LIHC (Fig. 1G). Taken together, SPATS2 was significantly correlated with clinicopathological parameters of patients with HCC.

**Fig. 2:**
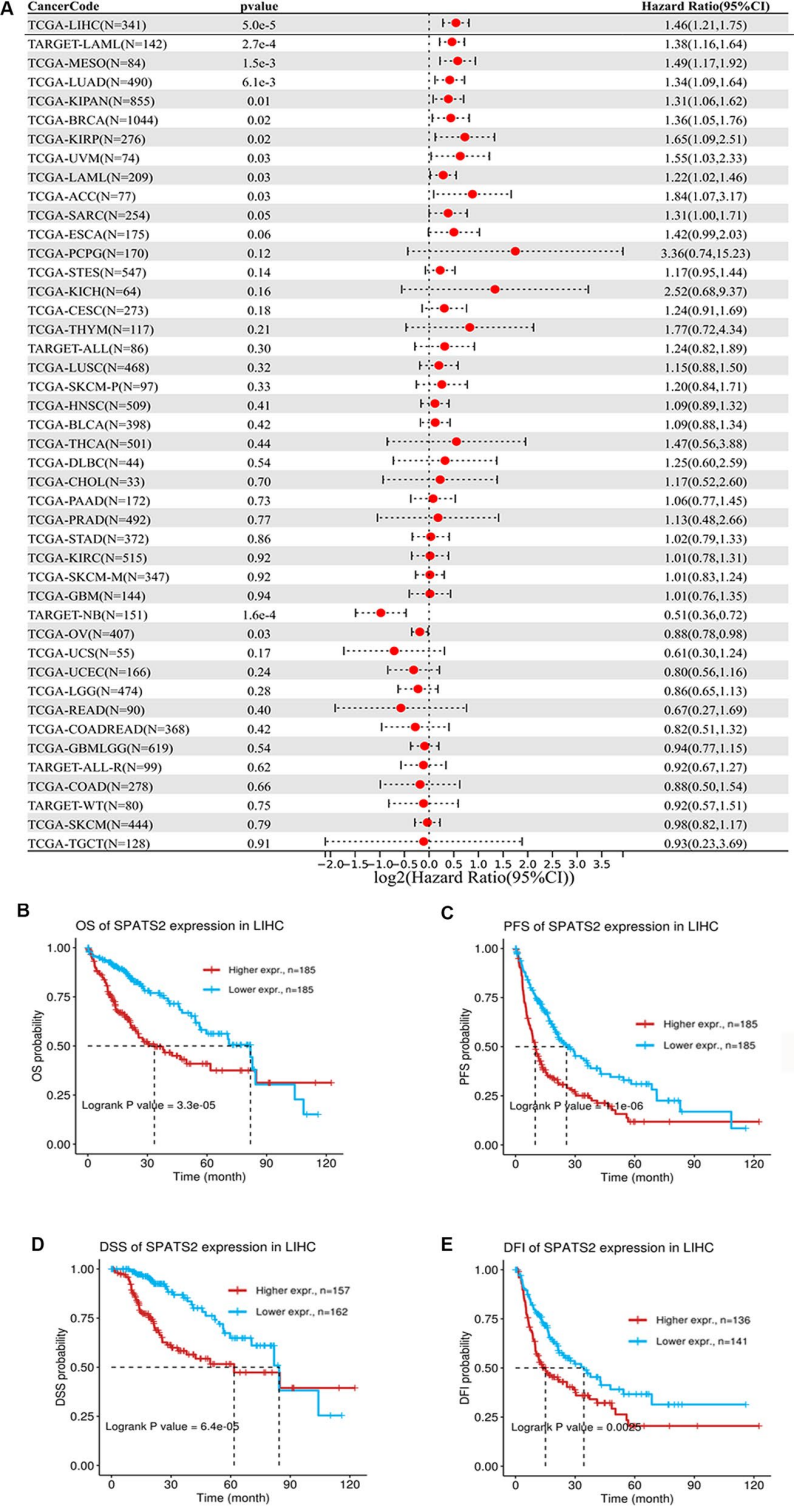
Prognostic value of SPATS2 in patients with HCC. **A** Survival map of SPATS2 gene with significant associations in pan cancer. **B**–**E** The high expression group of SPATS2 has significantly worse OS (**B**), PFS (**C**), DSS (**D**), and DFI (**E**) than the low expression group in HCC. *p* < 0.05 is considered statistically significant

To investigate the association of SPATS2 expression with prognosis, the survival association analysis was performed in pan cancer. As shown in Fig. 2A, overexpression of SPATS2 was related to poor prognosis in most cases, including LIHC. Moreover, Kaplan–Meier plotter tool showed that the elevated SPATS2 was drastically associated with a shorter OS, PFS, DSS, and DFI in patients with HCC (Fig. 2B–E). These results suggested that SPATS2 has a good prognostic evaluation value in the whole processes of liver cancer development.

### Functional enrichment analysis of SPATS2 and its co-expressed genes in HCC

**Fig. 3:**
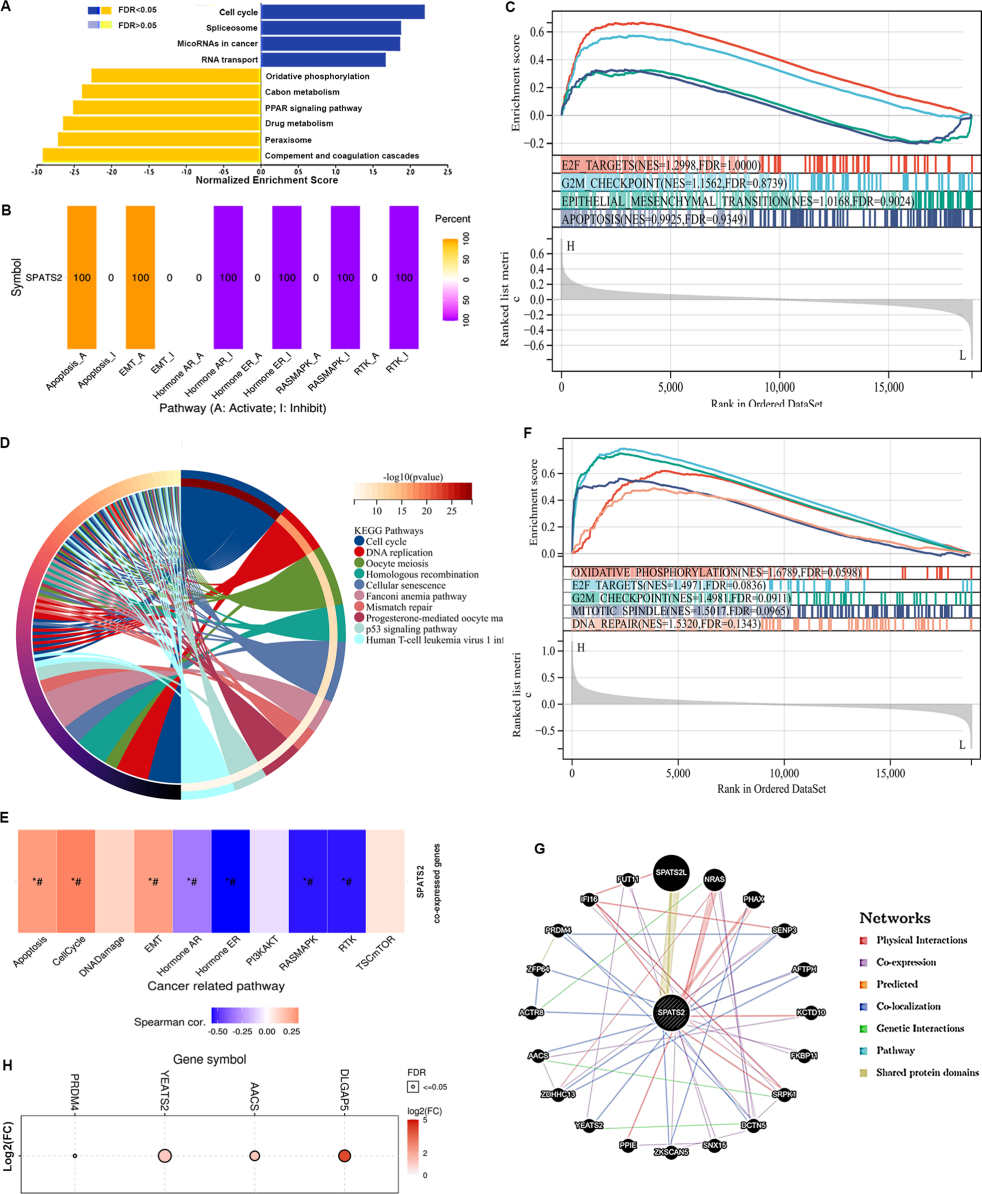
Enrichment analysis of SPATS2 and its co-expressed genes in HCC. **A** The enrichment analysis based on TCGA_LIHC data. Representative GO terms of SPATS2 in LIHC. **B** The enrichment analysis of SPATS2-related pathways in LIHC. Red represents positive correlation; blue represents negative correlation. **C** GSEA analysis the enrichment of signature genes involved in E2F target, G2M checkpoint, EMT, and apoptosis in SPATS2 high or low expression group. **D** The KEGG function enrichment analysis of SPATS2-related genes in LIHC. **E** The biological pathways of SPATS2 co-expressed genes in HCC. Red represents positive correlation; blue represents negative correlation. * indicates *p* value ≤ 0.05; # represents FDR ≤ 0.05. *p* value < 0.05 is considered statistically significant. **F** GSEA analysis the enrichment of signature genes involved in E2F target, G2M checkpoint, Mitotic spindle, and DNA repair in DLGAP5 high or low expression group. **G** SPATS2 interacting proteins are predicted through GeneMANIA database. **H** The expression of SPATS2 interacting proteins (DLGAP5, YEATS2, AACS and PRDM4) in HCC

To further explore the biological function of SPATS2 in HCC, we performed KEGG pathway analysis based on the TCGA-LIHC data. As shown in Fig. 3A, the top three significantly enriched pathways of SPATS2 included cell cycle, spliceosome, and MicoRNAs in HCC cancer. Furthermore, pathways analysis via GSCA indicated that SPATS2 expression was markedly correlated with apoptosis, and EMT; whereases negatively associated with hormone, RASMAPK and RTK pathways (Fig. 3B). GSEA results also showed that SPATS2 was positively associated with cell cycle, EMT and apoptosis (Fig. 3C). Therefore, SPATS2 plays an important role in cell cycle, cell apoptosis, and cancer cell metastasis processes in HCC.

Next, we obtained the SPATS2 mRNA expression-associated genes in liver cancer. In total, 401 positively (R > 0.5) and 159 negatively (R < − 0.5) correlated gene were found, and the top 50 related genes were displayed on the heat map (Additional file 1: Fig. S1). These genes were significantly enriched for cancer-promoting terms, such as cell cycle and DNA replication (Fig. 3D). Furthermore, GSCA pathways enrichment analysis results showed that these genes were also positively correlated with apoptosis, cell cycle and EMT pathways in LIHC (Fig. 3E).

Subsequently, these pathways related genes were obtained from GSEA database and PPI networks were constructed. In total 550 SPATS2 significantly related genes, we only found that SPATS2 directly interact with DLGAP5. GSEA result indicated that DLGAP5 was also enriched in the oxidative phosphorylation, cell cycle, and DNA repair pathways (Fig. 3F). In addition, the interacted proteins of SPATS2 were obtained based on the GeneMANIA database (Fig. 3G). YEATS2, AACS and PRDM4 were co-localization proteins of SPATS2. These genes were up-regulated in LIHC (Fig. 3H). These results indicated that SPATS2 maybe regulate these genes expression to affect cell apoptosis, cell cycle, and invasion processes in HCC progression.

### Knockdown of SPATS2 affects cell cycle, apoptosis and invasion of HCC cells

**Fig. 4:**
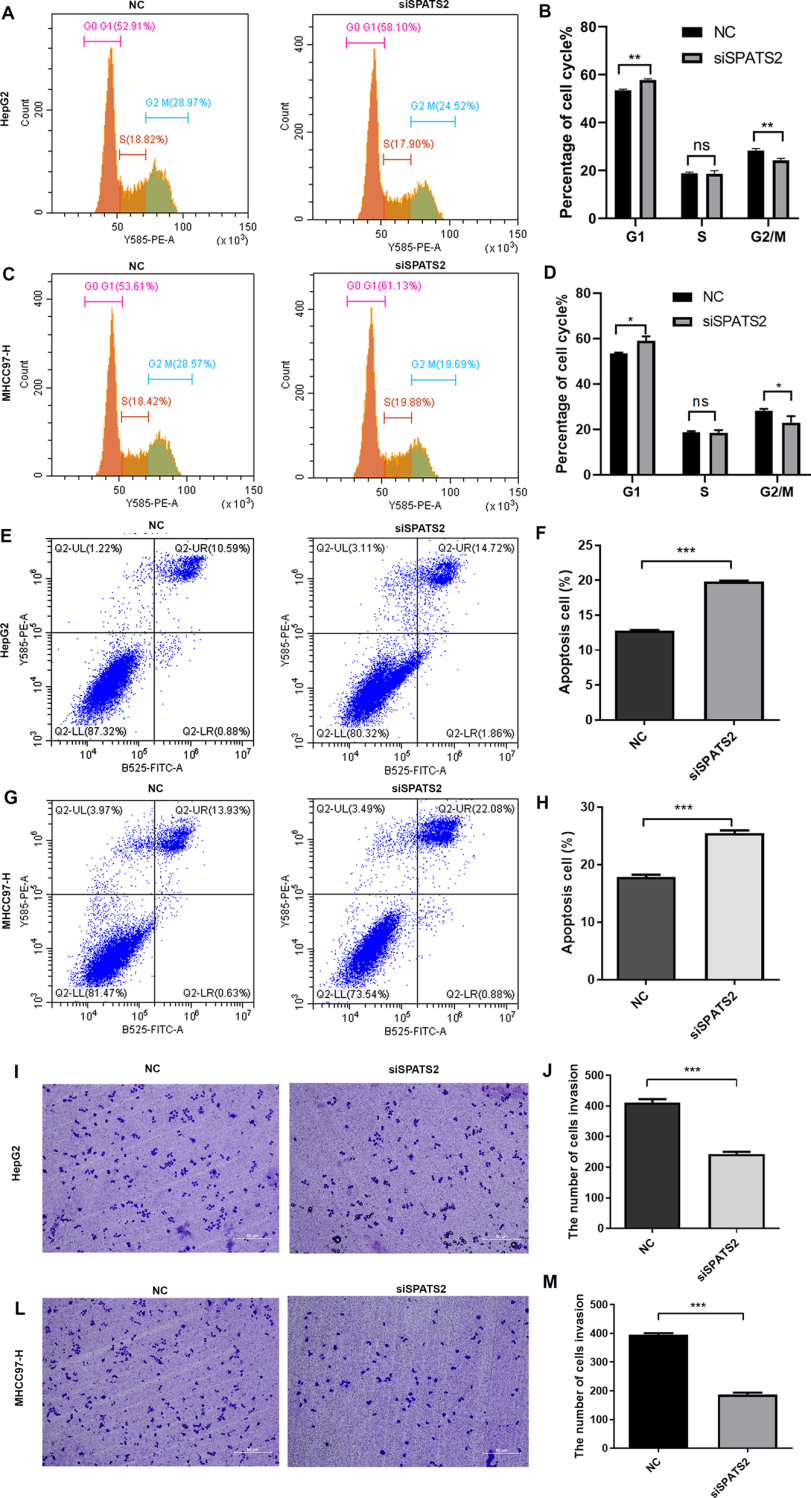
Knockdown of SPATS2 affects cell cycle, apoptosis and cell invasion in HCC cells. **A**–**D** The flow cytometry was performed to analyze the cell cycle distribution of HepG2 and MHCC97-H cells after SPATS2 down-regulation. **E**–**H** The flow cytometry was performed to analyze the cell apoptosis of HepG2 and MHCC97-H cells after SPATS2 down-regulation. **I**–**M** Transwell assays were performed to detect the migration of HepG2 and MHCC97-H cells after SPATS2 down-regulation. *p < 0.05, **p < 0.01, **P < 0.001

To further investigate the functions of SPATS2 in HCC progression, the siRNAs was transfected in HepG2 and MHCC97H cells to silence SPATS2 mRNA expression. Hereafter, we examined cell cycle and apoptosis progression in HCC cell lines by flow cytometry. The results showed that knockdown of SPATS2 significantly increased the percentage of cells in the G0/G1 phase and decreased the G2/M phase, suggesting that SPATS2 may regulate the cell cycle in liver cells (Fig. 4A–D). Moreover, the downregulation of SPATS2 reduced apoptosis of HCC cells ((Fig. 4E–F). In addition, knockdown of SPATS2 dramatically inhibited the invasion ability of HCC cells (Fig. 4I–M). Therefore, SPATS2 plays an important role in HCC progresses.

### The expression of SPATS2 is upregulated by epigenetic modification in HCC

**Fig. 5:**
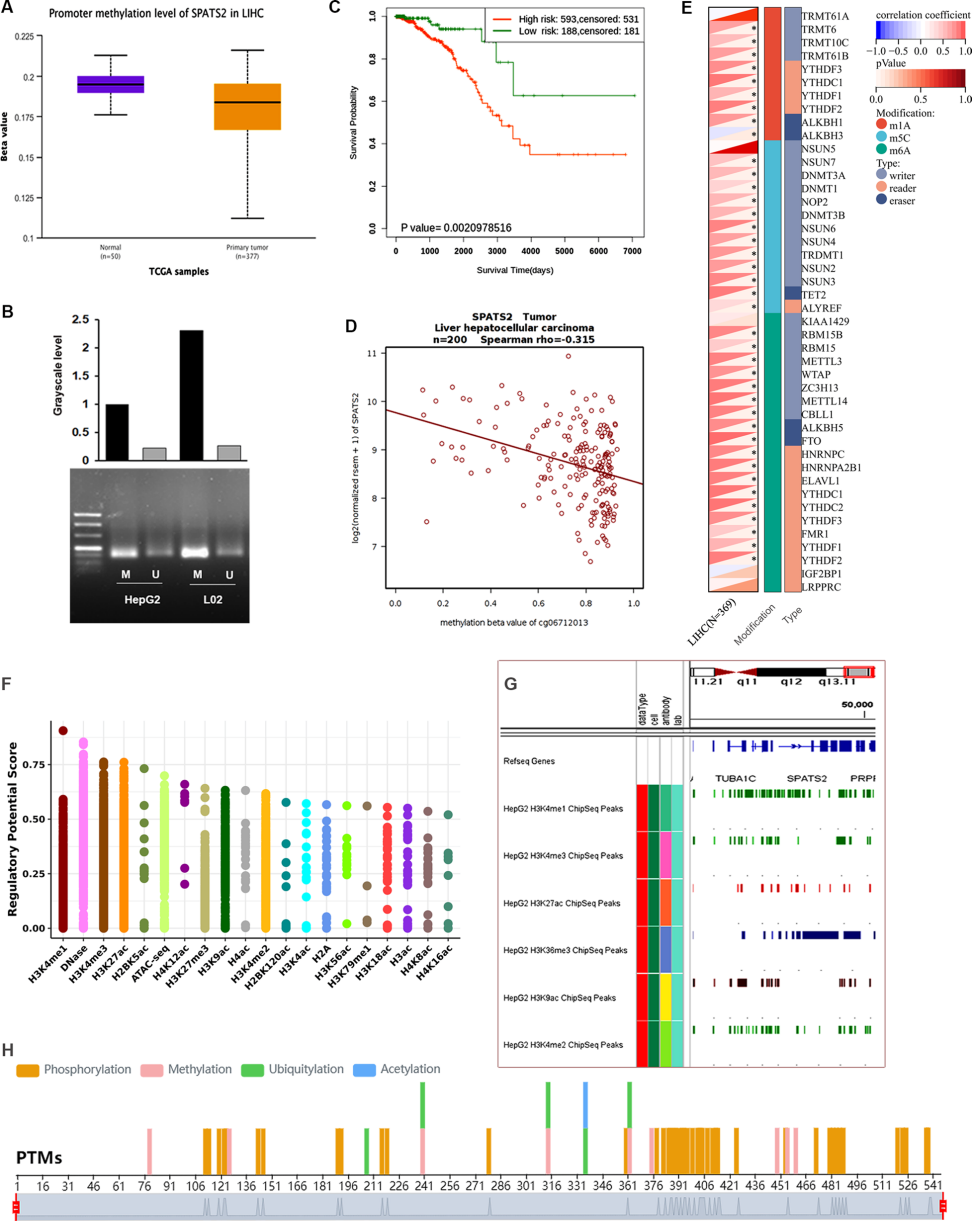
Comparison of epigenetic alterations associated with SPATS2 in HCC. **A**–**D** Correlation between SPATS2 mRNA expression and its methylation levels. **A** Promoter methylation level of SPATS2 is lower in primary tumor than normal tissue. **B** Promoter methylation level of SPATS2 is decreased in HepG2 cells. **C** The overall survival was compared between patients with high or low methylation of SPATS2. **D** The correlation between SPATS2 expression and DNA methylation level. **E** The correlation between the expression of SPATS2 mRNA and m6A methylation regulatory factors in HCC. Correlations are depicted with Spearman’s rho values and statistical significance. **F**–**H** Histones epigenetic alterations in SPATS2 promoter. **F** Histones epigenetic regulatory potential in SPATS2 mRNA expression. **G** H3K4me1, H3K4me3, H3K27ac, H3K9ac were found enrichment in SPATS2 promoter in HepG2 cell. **H** Methylation and acetylation sites in SPATS2 protein

It is well known that epigenetic regulation plays an important role in gene mRNA expression. DNA methylation level of SPATS2 was assessed in HCC. We found that SPATS2 was similarly unmethylated in HCC samples (Fig. 5A). MSP assay indicated that the methylation level was decreased in HepG2 cells (Fig. 5B). Moreover, high risk was observed in low methylation group (Fig. 5C). The expression of SPATS2 was negatively associated with its methylation level in HCC (Fig. 5D).

Considering that m6A methylation in tumorigenesis and development. Then, we explored the relationship between the expression of SPATS2 mRNA and m6A methylation in LIHC. The heatmap indicated that SPATS2 mRNA was positively correlated with most m6A methylation regulatory factors (Fig. 5E). In addition, we also investigated the histone modification in SPATS2 promoter region. As shown in Fig. 5F, H3K4me1, H3K4me3, H3K27ac, and H3K36me3 modifications that promote gene expression were significantly enriched in the promoter region of SPATS2 gene (Fig. 5G). Additionally, multiple acetylation and methylation sites were found in SPATS2 (Fig. 5H). Taken together, these data suggested that epigenetic alterations play an important role in regulating the abnormal expression of SPATS2 in LIHC.

### Correlations between SPATS2 expression and immune infiltration in HCC

**Fig. 6:**
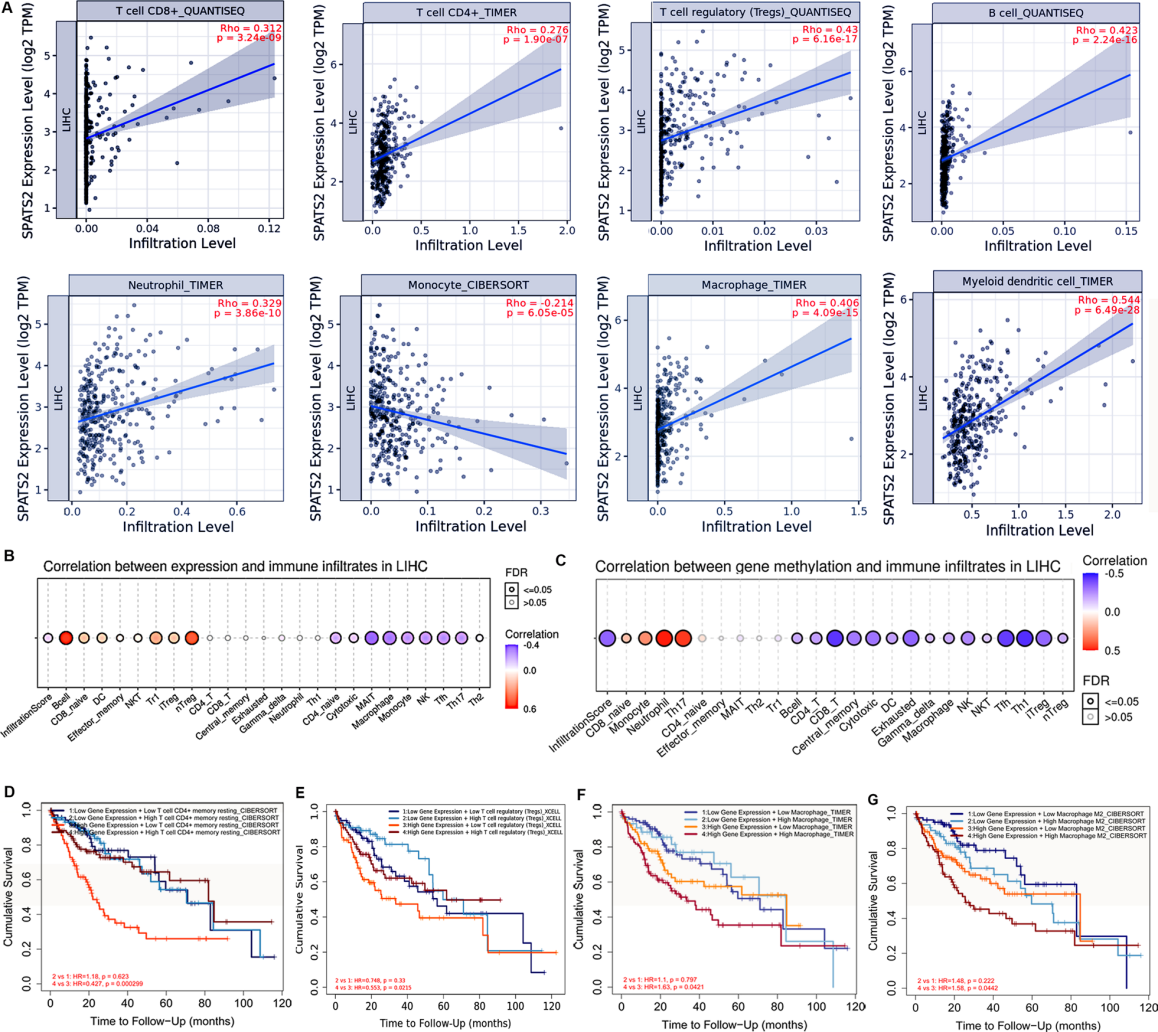
Relationship between immune cell infiltration and SPATS2 level in HCC. **A** Correlation of SPATS2 expression with immune cells infiltration levels in HCC. **B**, **C** Correlations of SPATS2 expression (**B**) and methylation (**C**) with infiltration levels of different immune cell subtypes in HCC. Red represents positive correlation; blue represents negative correlation. **D**–**F** Comparison of Kaplan–Meier survival curves of the high and low expression of SPATS2 in HCC based on immune cell subgroups, including CD4+T and memory resting cell (**D**), Tregs (**E**), macrophages (**F**), and macrophages M2 (**G**)

To further reveal the functions of SPATS2 in the whole processes of liver cancer development, the relationships between SPATS2 expression and immune cells in tumor microenvironment were investigated in LIHC. TIMER results indicated that there was a statistically positive correlation between SPATS2 mRNA expression and most of immune cells, such as T cells, B cells, Macrophage, Neutrophil, and Dendritic cells (Fig. 6A). Among of them, SPATS2 level was significantly correlated with Tregs, B cell, macrophage, and DC cells (rho > 0.35, *p* < 0.05) in LIHC, especially. Whereas, although it has significant relationship with the CD8+ , CD4+ , and neutrophil cells, the correlation coefficients were lower in LIHC. Additionally, GSCA database results indicated that SPATS2 expression was also positively associated with B cell and nTreg (cor > 0.4, *p* < 0.05) (Fig. 6B). Interestingly, methylation level of SPATS2 was significantly negatively associated with most of immune cells based on the GSVA results (Fig. 6C). In total, 15 of 24 immune cells were found that have a relationship with SPATS2 methylation. Specifically, the methylation of SPATS2 has a significant association (Cor < − 0.4, *p* < 0.05) with immune cell CD8_T, Tfh, and Th1cells. Therefore, SPATS2 maybe involve in anti-tumor immune response to regulate the progression of HCC.

To further investigate whether the expressions of SPATS2 affected prognosis of patients with HCC is partly attributed to immune cells infiltration, a prognosis analysis was performed via the Kaplan Meier plotter based on the mRNA level of SPATS2 and the immune cells infiltration in HCC. The prognosis of SPATS2 associated immune cells was evaluated in LIHC. The results showed that the high level of SPATS2 with low CD4+T and memory resting cells or Tregs cells infiltrating indicated a worse prognosis in patients with HCC (Fig. 6D, E); the SPATS2 expression with high level of macrophage cells infiltration is associated with a worse prognosis in patients with HCC, especially M2 macrophage cells (Fig. 6F, G). However, other immune cells infiltration combining with high SPATS2 level did not show significant associations in prognosis of HCC patients. Taken together, SPATS2 overexpression may affect prognoses of patients with HCC in part due to immune cells infiltration.

### Expression of SPATS2 in immune cells based on the HCC single-cell sequencing data

**Fig. 7:**
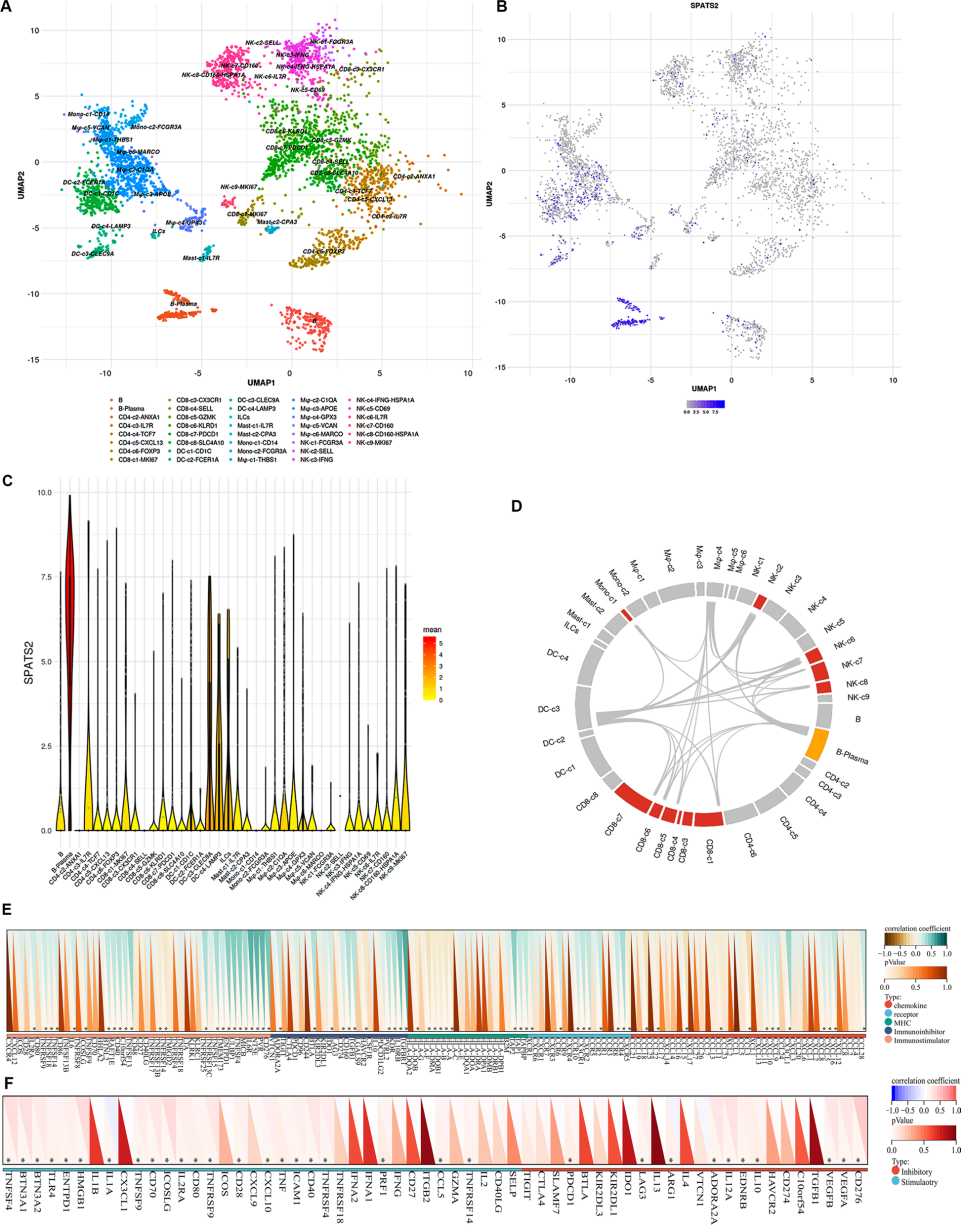
Single-cell RNA-seq analysis of SPATS2 in the tumor tissue of HCC patients. **A** The t-SNE map depicting clusters of immune cells in the tumor tissues and adjacent tissues of five HCC patients. **B** Expression t-SNE maps for the SPATS2 in the tumor tissues and adjacent tissues of HCC. **C** The iolin plots showing the distribution of SPATS2 in various immune cell clusters. **D** Circle plot illustrating the Interaction between B plasma and other immune cells. **E** Heat maps show that the expression of SPATS2 has a significantly positive correlation with immune related genes, including inhibitory and stimulatory, immune chemokine, receptor, MHC. **F**. Heat maps display a correlation between SPATS2 expression and immune checkpoints. *p < 0.05 was considered statistically significant

In order to further elucidate the function of SPATS2 in immune infiltration, single-cell analysis was performed to assess the expression of SPATS2 in immune cells of HCC patients. As shown in Fig. 7A, the tSNE maps demonstrated that 38 immune cell types were annotated. By comparing tumor with adjacent tissue, we found that SPATS2 level was widely expressed in B cells, macrophage, and DC cells (Fig. 7B, C). Moreover, cell interaction results showed that B plasma has a correlation with DC, NK and macrophages cells in HCC patients (Fig. 7D). It is consistent with result that SPATS2 is positively associated with B cells, macrophage, and DC cells. Therefore, SPATS2 likely plays an important role in tumor immune response to regulate the functions of these immune cells.

### Relationship between SPATS2 and immune-related genes in HCC

As the expression of SPATS2 showed a significant association with immune cell infiltration, we evaluated the correlations between the expression of SPATS2 and immunomodulators and the infiltration level in HCC. As shown in Fig. 7E, SPATS2 expression was significantly correlated with immune-related inhibitory and stimulatory. Moreover, thirteen chemokine-related genes, nine MHC-related genes and receptor-related genes were associated with SPATS2 expression (Fig. 7E). Furthermore, the relationships between the SPATS2 expression and the most common immune checkpoints were analyzed. The expression of SPATS2 was positively correlated with the most of immune checkpoints in LIHC (Fig. 7F). These findings further supported the result that SPATS2 is critical in immune regulation of HCC.

## Discussion

In recent years, SPATS2 has been reported to contribute to the tumorigenesis of multiple malignancies, including liver cancer [6, 12]. However, the potential role of SPATS2 in HCC is yet to be elucidated, especially in tumor immune microenvironment. To further validate the function of SPATS2 in HCC, we comprehensively analyzed gene expression, prognosis, epigenetic regulation, and tumor immune cells infiltration of SPATS2 in HCC. In the present study, SPATS2 was determined to be upregulated in HCC. Furthermore, high expression of SPATS2 was indicative of an unfavorable clinicopathological feature and poor prognosis in patients with HCC. Moreover, we found that SPATS2 dramatically promotes proliferation and invasion of HCC cells. Consistent with previous results, SPATS2 expression acts as an oncogene, which could be served as a diagnostic and prognostic biomarker in liver cancer. In addition, biological pathway and functional enrichment analysis in our present study illustrated that SPATS2 likely regulates cell cycle, apoptosis, and EMT in HCC. Furthermore, we explored that SPATS2 co-expressed genes were also enriched for cell cycle, DNA replication, apoptosis, and EMT in HCC. Functionally, our results indicated that knockdown of SPATS2 likely dampened HCC development and metastasis by regulating cell cycle and apoptosis.

It is well known that the immune system plays an important role in controlling cancer progression. Recent year, immunotherapy has developed as the new first-line treatment option for advanced HCC [4, 21, 22]. Previous study indicated that tumor immune cell infiltration generates an immunosuppressive TME and presences a generally correlations with a worse prognosis [23, 24]. In tumor infiltrated immune cells, DCs are a unique class of immune cells that act as a bridge between innate and adaptive immunity, which plays a critical role in generating anti-tumor CD8 T cell immunity [25]. Treg cells has been reported that the they could inhibit the killing ability of CD8+T cells and results in a poor prognosis in HCC [24, 26]. In our study, SPATS2 was found for the first time to be highly positively correlated with the tumor infiltration of Tregs, B cell, macrophage, and DC cells in HCC. Moreover, the single cell data of HCC showed that SPATS2 was specifically expressed in B cells, macrophage, and DC cells in HCC. Thus, SPATS2 plays an important role in the recruitment and regulation of some immune infiltrating cells in liver cancer. Furthermore, we also confirmed that SPATS2 combined with CD4+ , Tregs, or macrophage infiltration level was associated with prognosis in HCC. Taken together, we hypothesized that SPATS2 expression could lead to HCC progression and poor prognosis by affecting the infiltration of above immune cells.

Epigenetic regulation was involved in the development and progression of a variety of tumors. We found that the expression of SPATS2 was significantly negatively correlated with the average methylation level of the promoter, suggesting that DNA methylation may be one of the mechanisms for its up-regulation. Moreover, our results showed that DNA methylation of SPATS2 was significantly negatively correlated with the infiltration of immune cells. SPATS2 was significantly negatively associated with the CD8 T, Th1, and Thf cells, positively correlated with the neutrophil and Th17 cells in HCC. It has been confirmed that the major reasons for tumor escape in the immune system include the dysfunction of CD8+T cells and the presence of excessive suppressor T cells [26]. More research results have established the function of CD8+T cells in the formation and progression of HCC, including diagnosis/treatment/prognosis [27]. Moreover, targeting CD8+T cells is the main direction of immunotherapy for HCC [28]. The neutrophils cells are significantly associated with cancer progression and metastasis [29]. It also mainly suppresses antitumor immunity by inducing apoptosis of CD8+T cells through nitric oxide production-mediated TNF-α [30]. Furthermore, we found that SPATS2 are positively correlated with the expression of multiple immune checkpoints. Therefore, these studies suggest that SPATS2 plays an important role in occurrence and development of tumors and contribute to the development of immunotherapy therapies. SPATS2 may be a novel target gene for immunotherapy in HCC. However, its exact mechanism needs to be further confirmed.

In conclusion, our present study explored that SPATS2 was significantly correlated with cancer progression, poor survival, epigenetic regulation and immune infiltration in patients with HCC. Moreover, SPATS2 was associated with increased immune cellular infiltration and the expression of immune checkpoints. Therefore, SPATS2 may be an important factor in the progression of HCC. The present study helps us to elucidate the significance of SPATS2 in HCC progression, especially in tumor immune microenvironment. These results will provide a new theoretical basis for targeted therapy in HCC.

## Supplementary Information

**Additional file 1**: **Fig. S1**. The co-expresed genes of SPATS2 in LIHC.

## Data Availability

The datasets used in this article are publicly available as described in Materials and Methods. All data generated during this study are included in this published article and its supplementary information files. The data available in TCGA (https://portal.gdc.cancer.gov/) databases. The datasets analyzed during the current study available from the corresponding author on reasonable request.
