# Supplementary material for: SPATS2 is correlated with cell cycle progression and immune cells infiltration in hepatocellular carcinoma
Source: BMC Gastroenterol. 2023 Jan 11;23:8. doi: 10.1186/s12876-022-02633-y (PMC9832668; doi:10.1186/s12876-022-02633-y)

**Figure S1.** **The SPATS2 correlated expression genes in LIHC.** (A, B) Heatmaps of the top 50 genes positively and negatively correlated with SPATS2 in LIHC, respectively. Red represents positive correlation; blue represents negative correlation.
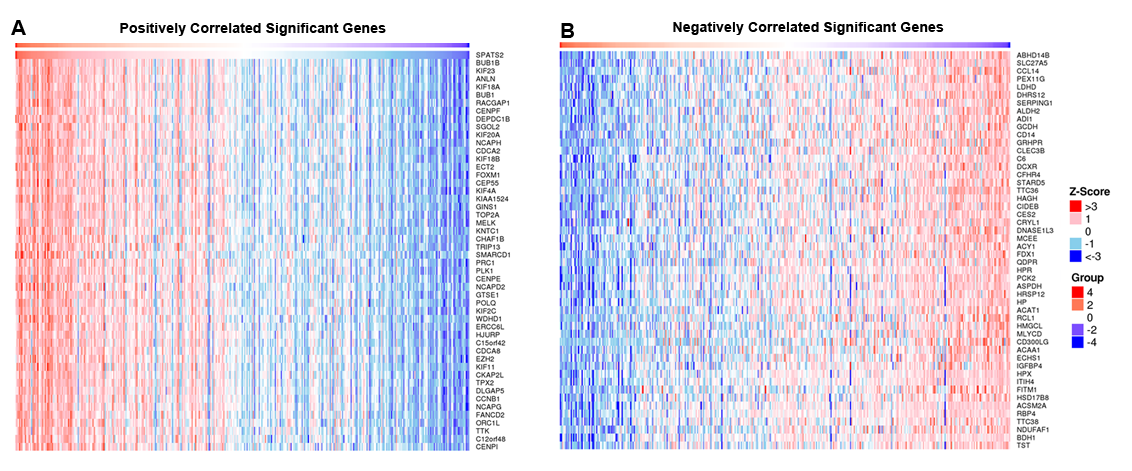

Supplement: Supplementary file 1 — Additional file 1: Fig. S1. The co-expresed genes of SPATS2 in LIHC. [file 12876_2022_2633_MOESM1_ESM.docx]
